# Substance Use and Delirium Trends in the Critically Ill During the Peri-COVID Period

**DOI:** 10.1097/CCE.0000000000001417

**Published:** 2026-05-19

**Authors:** Emma Bick, Benjamin J. Sines, C. Adrian Austin

**Affiliations:** 1 Department of Medicine, University of North Carolina, Chapel Hill, NC.; 2 Department of Pediatrics, University of North Carolina, Chapel Hill, NC.; 3 Division of Pulmonary and Critical Care Medicine, University of North Carolina, Chapel Hill, NC.; 4 Division of Geriatric Medicine, University of North Carolina, Chapel Hill, NC.

**Keywords:** COVID-19, delirium, intensive care unit, interrupted time series, substance use

## Abstract

**IMPORTANCE:**

Substance use complicates many hospitalizations by increasing delirium rates, acuity, and costs. Its prevalence rose during the COVID-19 pandemic, potentially contributing to increased delirium in hospitalized patients.

**OBJECTIVES:**

To describe trends in substance use and delirium complicating ICU and floor admission around the COVID-19 pandemic and determine whether increased societal substance use was associated with increased prevalence of delirium and substance use in the critically ill.

**DESIGN, SETTING, AND PARTICIPANTS:**

Retrospective cohort study using a large deidentified database of patients admitted to the medical ICU and medicine wards at a single U.S. academic center. We used *International Classification of Diseases*, Tenth Revision (ICD-10) codes for substance use and a validated ICD-based delirium phenotype to identify exposures.

**MAIN OUTCOMES AND MEASURES:**

Monthly prevalence of delirium and substance use was calculated in the ICU and on the floor. We performed interrupted time series analysis to determine the effect of the COVID-19 pandemic on these trends.

**RESULTS:**

Pre-pandemic, ICU admissions complicated by substance use were declining. The onset of COVID-19 marked a significant reversal, with stepwise continued increase upon entering the recovery phase of the pandemic. Floor admissions complicated by substance use were gradually decreasing pre-pandemic, remained steady during lockdowns, and rose in the recovery phase. ICU delirium prevalence was decreasing pre-pandemic but rose abruptly with the onset of lockdowns, then declined during the pandemic. Delirium prevalence on the floor was increasing before the pandemic and remained stable during the peak and has continued to rise into the recovery phase.

**CONCLUSIONS AND RELEVANCE:**

Post-COVID, substance use increased in both ICU and floor admissions. Delirium increased during COVID but has since decreased in the ICU. Prevalence has continued to rise on the floor. Substance use and delirium trends during the COVID-19 pandemic reflect strain on the healthcare system during this time.

**KEY POINTS**

**Question:** How has the COVID-19 pandemic affected the prevalence of substance use and delirium complicating ICU and floor admissions?

**Findings:** In this retrospective cohort study of 240,913 adult admissions (7,596 ICU; 233,317 floor), ICU delirium prevalence rose sharply with COVID-19 onset but declined afterward, whereas floor delirium continued to increase into recovery. Substance use prevalence, which was decreasing pre-pandemic, rose significantly post-COVID in both ICU and floor patients.

**Meaning:** COVID-19 altered patterns of substance use and delirium in hospitalized patients, underscoring the need for ongoing screening and prevention strategies.

Substance use disorder, per the *Diagnostic and Statistical Manual of Mental Disorders: DSM-5* has a criteria-based definition including impaired control over substance use, social impairment, risky use, tolerance, and withdrawal, encompassing multiple substances including opioids and alcohol (1). Over the past few decades, rates of substance use have been increasing in the United States (2, 3). Substance use disorder is common among hospitalized adults and accounts for up to one-third of adult inpatient admissions and is associated with increased hospital length of stay and illness complexity (4).

Substance use is also associated with increased incidence of delirium (5). Delirium, an acute change in attention as a result of an acute insult, is associated with multiple negative patient outcomes, including increased in-hospital mortality, longer length of stay in both the ICU and in the hospital, decreased likelihood of returning home after hospitalization, and decreased likelihood of return to prior functional and cognitive baseline (6–16).

The COVID-19 pandemic created a societal environment that had a profound negative impact on mental health, including a rise in substance use. Social isolation and the increased strain on already limited mental health services during the pandemic led to increased rates of substance use (17). Consequently, during the COVID-19 pandemic, the United States saw a more rapid increase in the already rising rates of alcohol and drug-related deaths (17).

We hypothesized that the increased rate of societal substance use during the pandemic would correlate with increased rates of hospitalizations complicated by substance use. The objective of this study was to describe temporal trends in hospital and ICU admissions complicated by substance use before, during, and after the COVID-19 pandemic period and determine if there was a corresponding change to the prevalence of ICU delirium over this same period. Our hypothesis was that we would see an increase over pre-COVID baseline in both substance use and delirium prevalence during the peak COVID-19 years (2020–2021) with a continued high prevalence in 2022.

## MATERIALS AND METHODS

We performed a retrospective cohort study using data collected on patients admitted to University of North Carolina Hospital at Chapel Hill (UNC) between January 1, 2018 and December 31, 2022. The UNC institutional review board (IRB) reviewed and approved this study (UNC IRB Number 23-0160, approved January 24, 2023). All procedures were followed in accordance the Helsinki Declaration of 1975. We used a large deidentified database of all UNC admissions to identify all adult patients (age older than 17 yr) admitted to the medical ICU (MICU) and medicine acute inpatient teams.

We further stratified these cohorts by the presence of documented delirium and/or coma and substance use during their index hospitalization. We identified delirium using *International Classification of Diseases*, Tenth Revision (ICD-10) diagnosis codes based on a previously published and validated administrative claims-based phenotype for delirium as well as ICD-10 codes identifying coma (**Supplemental Content**, https://links.lww.com/CCX/B629) (18). This phenotype was developed and evaluated in hospitalized patients and performed reasonably well in identifying delirium based on ICD-10 codes compared with direct patient assessment (sensitivity 18%, specificity 98%) (18). Delirium identification in this cohort relied on diagnosis codes assigned during the hospitalization, which reflect documentation by the treating medical team and subsequent coding into the electronic medical record. Delirium was identified when diagnosed and documented by the treating clinicians.

Substance use was identified by ICD-10 codes for alcohol use (F10), opioid use (F11), cannabis use (F12), sedative, hypnotic, anxiolytic use (F13), cocaine use (F14), stimulant use (F15), hallucinogen use (F16), nicotine use (F17), inhalant use (F18), and other psychoactive use (F19) (Supplemental Content, https://links.lww.com/CCX/B629). These codes represent substance use diagnoses documented during the hospitalization and do not distinguish between active intoxication, withdrawal, or historical use. All analyses were also evaluated by year and inpatient level of acuity.

We calculated prevalence of documented substance use and delirium in medical admissions by month stratified by level of care. To better evaluate temporal changes, we performed an interrupted time series analysis to assess the trends of delirium and substance use before, during, and after COVID-19. We defined the “interruption” as the period of peak hospital admissions for COVID-19 in North Carolina associated with the declaration of a public health emergency in March 2020 (19). A run-in phase before this interruption spanned January 2018 to June 2020 and established a baseline for monthly prevalence of delirium and substance use in medical admissions. The “peak” period, captured the immediate effects of the pandemic, and was defined as August 2020 to February 2022. The “recovery” phase spanned April 2022 to December 2022 after the last major peak of COVID-19-related admissions and was expected to reflect a return to baseline hospital operations and societal conditions. Data were analyzed to identify changes in expected trends and patterns over these intervals. Prevalence trends for substance use and delirium were calculated using generalized least squares models. Autocorrelation in the data was ruled out with the Durbin-Watson test, and no further adjustments were made to the analysis. Statistical significance of step changes in prevalence of substance use or delirium and changes in trends over time before and after the COVID-19 pandemic-based lockdowns were assessed in generalized least squares models.

All analyses were performed in R Statistical Software (Version 4.3.3, R Core Team 2024; R Foundation for Statistical Computing, Vienna, Austria).

## RESULTS

Between 2018 and 2022, 233,317 patients were admitted to floor teams and 7,596 patients were admitted to the MICU (**Table 1**). A higher proportion of men had documented substance use in both the ICU and the floor compared with women (**Supplemental Tables 1–6**, https://links.lww.com/CCX/B629). The documented prevalence of delirium was similar between males and females in this time period (Supplemental Tables 1–6, https://links.lww.com/CCX/B629).

**TABLE 1.** Characteristics of Patients Admitted to the Medical ICU and Floor From 2018 to 2022

Clinical Demographic	2018–2022	
	ICU	Floor
*n*	7,596	233,317
Sex		
Male (%)	3,937 (51.8%)	114,405 (49.0%)
Female (%)	3,651 (48.1%)	118,780 (50.9%)
Unknown (%)	8 (0.1%)	132 (0.1%)
Age		
Younger than 18 yr	0	988 (0.4%)
18–64	3,838 (50.5%)	87,826 (37.6%)
65–84	3,108 (40.9%)	105,557 (45.2%)
85+	642 (8.5%)	38,825 (16.6%)
Unknown (%)	8 (0.1%)	121 (0.1%)
Race		
White/Caucasian	4,447 (58.5%)	160,087 (68.6%)
Black/African American	2,190 (28.8%)	56,233 (24.1%)
Asian/Pacific Islander	71 (0.9%)	1,701 (0.7%)
American Indian/Alaskan Native	86 (1.1%)	2,557 (1.1%)
Other	601 (7.9%)	9,961 (4.3%)
Refused/Unknown	201 (2.6%)	2,778 (1.2%)

### Substance Use Trends

**Figure 1.**
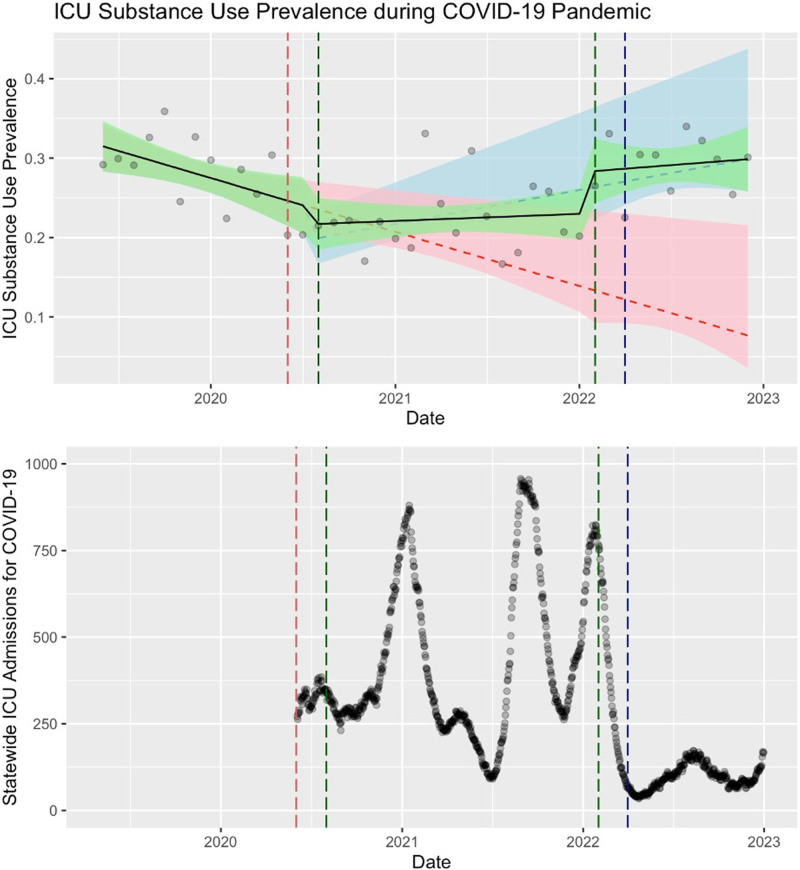
Prevalence of substance use complicating ICU admissions (2018–2022) overlaid on statewide ICU admissions for COVID-19 for North Carolina. *Green* is the observed results, *red* is a fitted estimate of the pre-pandemic trend if it were to continue forward, *blue* is a fitted estimate of the pandemic trend if it were to continue forward.

Before the COVID-19 pandemic, the monthly prevalence of ICU admissions with documented substance use showed a statistically significant downward trend (*p* value for trend, 0.013) (**Fig. 1**). With the increase of COVID-19 hospitalizations, the “interrupting event,” there was a reversal of the previous trend of declining prevalence and monthly prevalence of substance use in ICU admissions increased during peak COVID-19 periods, which was statistically significant (*p* value for change in trend, 0.024). In the “recovery” phase of the COVID-19 pandemic, there was a stepwise increase in documented substance use prevalence; however, the continued upward trend did not reach significance (*p* value for change in trend, 0.847).

**Figure 2.**
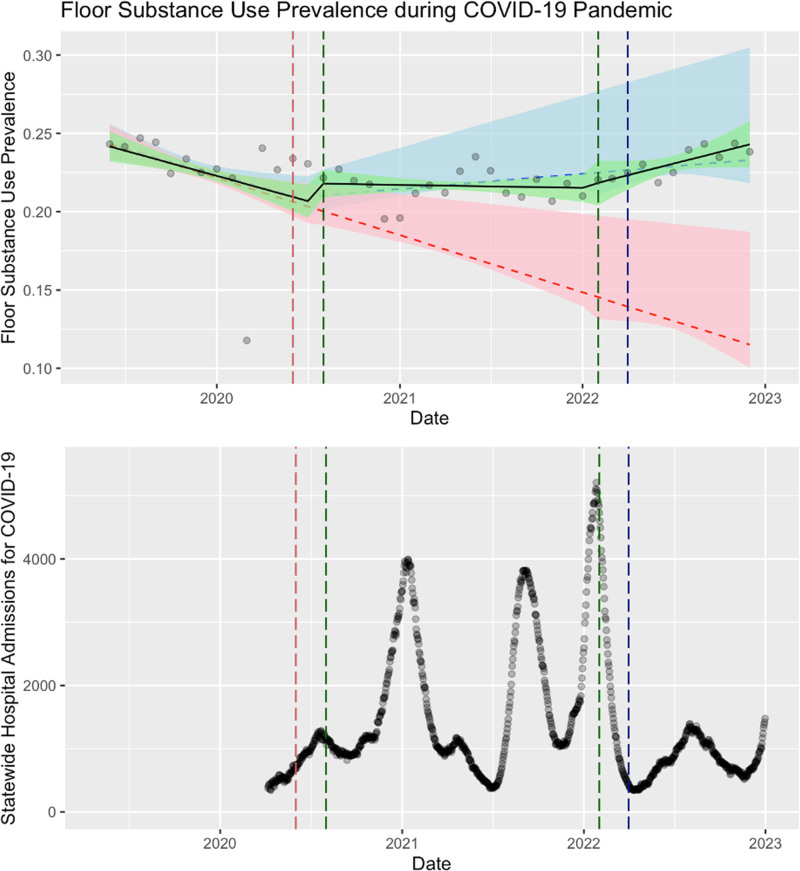
Prevalence of substance use complicating floor admissions (2018–2022) overlaid on statewide hospital admissions for COVID-19 for North Carolina. *Green* is the observed results, *red* is a fitted estimate of the pre-pandemic trend if it were to continue forward, *blue* is a fitted estimate of the pandemic trend if it were to continue forward.

On the floor teams, documented substance use prevalence declined significantly before the pandemic (*p* < 0.001) (**Fig. 2**). The beginning of the COVID-19 pandemic marked a statistically significant change in this trend with stable prevalence of substance use in floor team admissions (*p* = 0.021). During the “recovery phase,” prevalence of substance use began rising; a reversal of the pre-pandemic trends, which did not reach significance (*p* = 0.058).

### Delirium Trends

**Figure 3.**
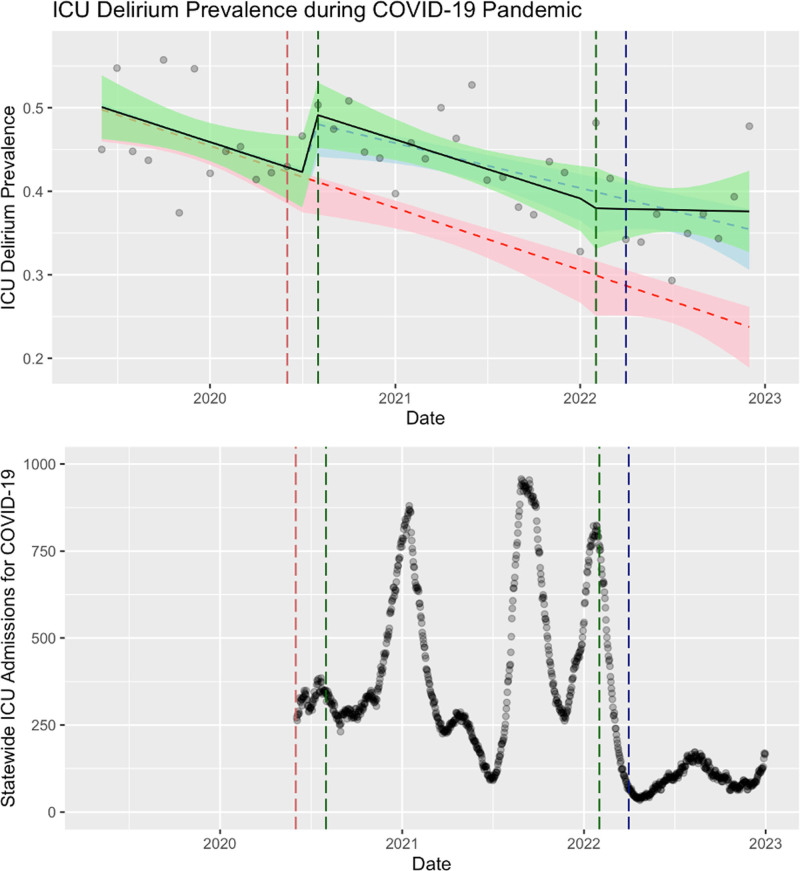
Prevalence of delirium complicating ICU admissions (2018–2022) overlaid on statewide ICU admissions for COVID-19 for North Carolina. *Green* is the observed results, *red* is a fitted estimate of the pre-pandemic trend if it were to continue forward, *blue* is a fitted estimate of the pandemic trend if it were to continue forward.

Before the pandemic, the prevalence of documented delirium in the ICU declined significantly (*p* = 0.029) (**Fig. 3**). With the onset of the COVID-19 pandemic and the initiation of lockdown, there was an immediate stepwise increase in documented delirium prevalence in the ICU (*p* = 0.021). Subsequent monthly trends showed a similar rate of decline in delirium prevalence as in the pre-pandemic period, which did not reach significance (*p* = 0.972). In the “recovery” phase of the COVID-19 pandemic, ICU delirium prevalence did not return to the pre-pandemic baseline. The downward slope of the prevalence of ICU delirium is flatter than before the pandemic (*p* = 0.245).

**Figure 4.**
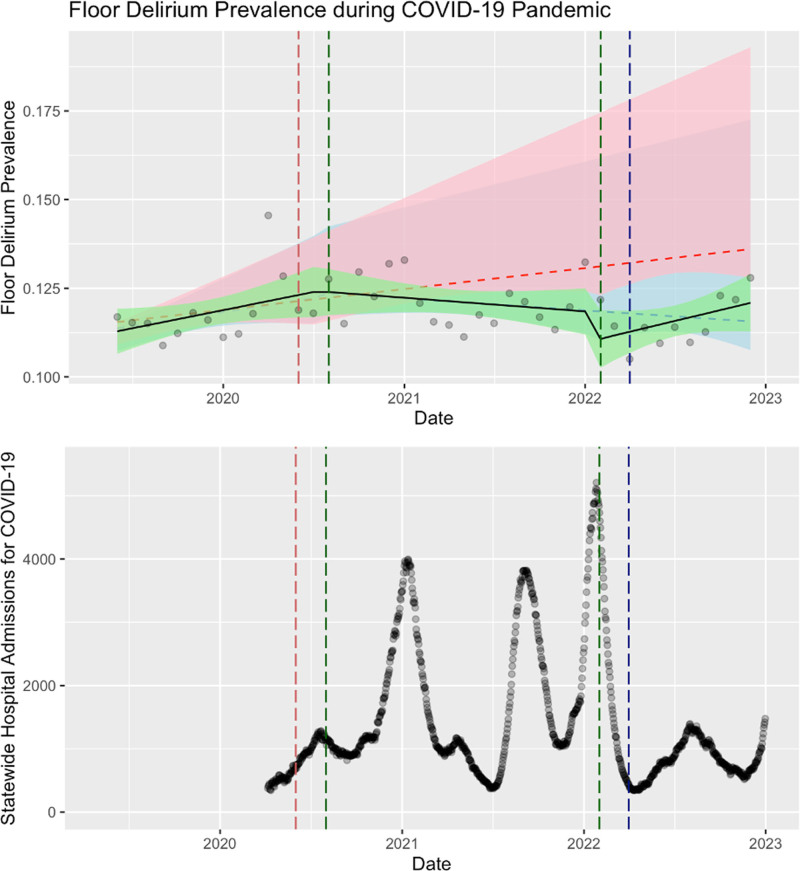
Prevalence of delirium complicating floor admissions (2018–2022) overlaid on statewide hospital admissions for COVID-19 for North Carolina. *Green* is the observed results, *red* is a fitted estimate of the pre-pandemic trend if it were to continue forward, *blue* is a fitted estimate of the pandemic trend if it were to continue forward.

Documented delirium prevalence on the floor was increasing before the COVID-19 lockdown (**Fig. 4**). With the onset of the COVID-19 pandemic, there was a significant decline in the slope of monthly prevalence of delirium (*p* = 0.040). In the “recovery” phase, delirium prevalence began to rise, although this did not reach significance.

## DISCUSSION

The three most striking findings of our study are: 1) the immediate increase in documented delirium in the ICU at the onset of the COVID-19 pandemic, 2) the subsequent gradual decline in ICU delirium prevalence during the pandemic and afterward, and 3) the increase in substance use prevalence in both floor and ICU settings in the post-COVID phase.

Before the pandemic, ICU delirium prevalence was decreasing. With the onset of COVID-19 lockdowns, we observed an immediate stepwise increase in documented ICU delirium followed by a return to a downward trend although at a slower rate than before the pandemic without an accelerated trendline to bring ICU delirium prevalence back to what would have been predicted without the pandemic effect. Substance use prevalence in the ICU and floor was decreasing before the pandemic. We observed an abrupt and ongoing increase in prevalence during the recovery phase of the pandemic in both the ICU and the floor. This suggests that the COVID-19 pandemic period was associated with noticeable shifts in documented prevalence of both delirium and substance use in hospitalized patients.

The increase in delirium prevalence at the onset of the pandemic reflects findings from other studies (20). This is likely attributable to higher levels of sedation used during COVID and due to increased severity of illness observed in the ICUs during this time period when ICU beds were primarily occupied by patients with severe respiratory failure. The declining prevalence of delirium over the course of the “peak” pandemic is likely due to resumption of delirium prevention best practices (i.e., A–F bundle practices) after the initial strain on the healthcare system. The finding that the rate of decline of delirium prevalence during the COVID period resembles the rate of pre-COVID decline demonstrates that best practice measures can have a measurable impact on delirium and reinforces the importance of their consistent implementation.

Perhaps our most striking finding is the rising prevalence of substance use in both the ICU and acute care setting during the post-COVID period. This increase aligns with broader societal trends of increased substance use during and after the COVID-19 pandemic (3). Although this study was not designed to evaluate outcomes associated with substance use in this population, we know that substance use is associated with increased morbidity and mortality in hospitalized patients, and its growing prevalence may further exacerbate complication rates and worsen outcomes both in the ICU and on general hospital floors (4, 5, 21). These trends underscore the need for routine substance use screening during an acute hospitalization as this interface with healthcare can provide an opportunity to intervene with robust resources to support recovery. Acute hospitalization often represents a pivotal moment when patients may be more open to intervention, offering a critical opportunity to address previously undiagnosed substance use disorders and encourage behavior change. Given these patterns, we can anticipate that substance use will continue to be a substantial contributor to morbidity in patients admitted to the hospital as well as the ICU.

A major strength of this study is the large sample size, comprising all patients admitted to our medicine teams and the MICU over 5 years, allowing for evaluation of trends in prevalence over distinct phases of the pandemic. However, reliance on medical record coding to identify patients is a notable limitation. Both delirium and substance use were identified using ICD-10 codes. Although we used a previously validated delirium phenotype, reliance on coding depends on clinician recognition and documentation. Previous data have shown the medical community is unreliable in identifying delirium in hospitalized patients, and it is routinely underrecognized (22, 23). Substance use may also be incompletely documented.

These limitations should be expected to bias toward underestimation of prevalence of substance use and delirium rather than overestimate, leading to likely conservative estimates. Although administrative databases cannot capture granular detail such as: sedation use, severity of delirium, intubated status, or substance use patterns, they allow for a reproducible method for large-scale data collection and trends across entire hospital systems and how these trends reflect broader national trends.

## CONCLUSIONS

These results highlight a shift in both delirium and substance use patterns in the peri-COVID time period. Decreasing prevalence of delirium in the ICU likely reflects the impact of broader adoption of ICU best practices. Post-pandemic trends show steadily increasing prevalence of substance use in patients admitted to the hospital. Assessing for a history of substance use in hospitalized patients with delirium is fundamental to effective management. These findings underscore the need for ongoing monitoring and tailored interventions to manage substance use and delirium in hospitalized patients.

## ACKNOWLEDGMENTS

The authors thank Agathe Ceppe of the University of North Carolina Hospital at Chapel Hill Marsico Lung Institute for her consultation and review of our statistical methods.

i2b2 software was used in conducting this study. i2b2 is the flagship tool developed by the i2b2 (Informatics for Integrating Biology and the Bedside) Center, the National Institutes of Health (NIH) funded National Center for Biomedical Computing based at Partners HealthCare System.

## Supplementary Material
