# Supplementary material for: Substance Use and Delirium Trends in the Critically Ill During the Peri-COVID Period
Source: Crit Care Explor. 2026 May 19;8(5):e1417. doi: 10.1097/CCE.0000000000001417 (PMC13189564; doi:10.1097/CCE.0000000000001417)
Supplement: Supplementary file 1 [file cc9-8-e1417-s001.pdf]

Table of Contents:

Page 2-5: Delirium ICD10 Codes

Page 6-13: Coma ICD10 Codes

Page 14-18: Alcohol Use ICD 10 Codes

Page 19-23: Opioid Use ICD 10 Codes

Page 24-26: Cannabis Use ICD 10 Codes

Page 27-33: Sedative, Hypnotic, Anxiolytic Use ICD 10 Codes

Page 34- 37: Cocaine Use ICD 10 Codes

Page 38-42: Stimulant Use ICD 10 Codes

Page 43-46: Hallucinogen Use ICD 10 Codes

Page 47- 48: Nicotine Use ICD 10 Codes

Page 49-52: Inhalant Use ICD 10 Codes

Page 53 -58: Other Psychoactive Substance Use ICD 10 Codes

Page 59: Table 1: Substance use and delirium/coma in the Medical Intensive Care Unit from 2018-2022

Page 59: Table 2: Substance use and delirium/coma in the Medical Intensive Care Unit from 2018-2022-- Males

Page 60: Table 3: Substance use and delirium/coma in the Medical Intensive Care Unit from 2018-2022-- Females

Page 60: Table 4: Substance use and delirium/coma on general medicine floors from 2018-2022

Page 61: Table 5: Substance use and delirium/coma on general medicine floors from 2018-2022 – Males

Page 61: Table 6: Substance use and delirium/coma on general medicine floors from 2018-2022 – Females

Delirium ICD10	
F05	Delirium due to known physiological condition
F10121	Alcohol abuse with intoxication delirium
F10221	Alcohol dependence with intoxication delirium
F10231	Alcohol dependence with withdrawal delirium
F10921	Alcohol use, unspecified with intoxication delirium
F11121	Opioid abuse with intoxication delirium
F11221	Opioid dependence with intoxication delirium
F11921	Opioid use, unspecified with intoxication delirium
F12121	Cannabis abuse with intoxication delirium
F12221	Cannabis dependence with intoxication delirium
F12921	Cannabis use, unspecified with intoxication delirium
F13121	Sedative, hypnotic or anxiolytic abuse with intoxication delirium
F13221	Sedative, hypnotic or anxiolytic dependence with intoxication delirium
F13231	Sedative, hypnotic or anxiolytic dependence with withdrawal delirium

F13921	Sedative, hypnotic or anxiolytic use, unspecified with intoxication delirium
F13931	Sedative, hypnotic or anxiolytic use, unspecified with withdrawal delirium
F14121	Cocaine abuse with intoxication with delirium
F14221	Cocaine dependence with intoxication delirium
F14921	Cocaine use, unspecified with intoxication delirium
F15121	Other stimulant abuse with intoxication delirium
F15221	Other stimulant dependence with intoxication delirium
F15921	Other stimulant use, unspecified with intoxication delirium
F16121	Hallucinogen abuse with intoxication with delirium
F16221	Hallucinogen dependence with intoxication with delirium
F16921	Hallucinogen use, unspecified with intoxication with delirium
F18121	Inhalant abuse with intoxication delirium
F18221	Inhalant dependence with intoxication delirium
F18921	Inhalant use, unspecified with intoxication with delirium
F19121	Other psychoactive substance abuse with intoxication delirium

F19221	Other psychoactive substance dependence with intoxication delirium
F19231	Other psychoactive substance dependence with withdrawal delirium
F19921	Other psychoactive substance use, unspecified with intoxication with delirium
F19931	Other psychoactive substance use, unspecified with withdrawal delirium
A812	Progressive multifocal leukoencephalopathy
E512	Wernicke's encephalopathy
G0430	Acute necrotizing hemorrhagic encephalopathy, unspecified
G0431	Post-infectious acute necrotizing hemorrhagic encephalopathy
G0432	Post-immunization acute necrotizing hemorrhagic encephalopathy
G0439	Other acute necrotizing hemorrhagic encephalopathy
G92	Toxic encephalopathy
G9340	Encephalopathy, unspecified
G9341	Metabolic encephalopathy
G9349	Other encephalopathy
I673	Progressive vascular leukoencephalopathy

I674	Hypertensive encephalopathy
I6783	Posterior reversible encephalopathy syndrome
J1081	Influenza due to other identified influenza virus with encephalopathy
J1181	Influenza due to unidentified influenza virus with encephalopathy
P9160	Hypoxic ischemic encephalopathy, unspecified
P9161	Mild hypoxic ischemic encephalopathy
P9162	Moderate hypoxic ischemic encephalopathy
P9163	Severe hypoxic ischemic encephalopathy

Coma ICD10	
E035	Myxedema Coma
G935	compression of brain
G936	cerebral edema
G9382	brain death
R400	somnolence
R401	stupor
R4020	unpspecified coma
R402110	coma scale, eyes open, never, unspecified
R402111	coma scale, eyes open, never, in the field
R402112	coma scale, eyes open, never, at arrival to the emergency department

R402113	coma scale, eyes open, never, at hospital admission
R402114	coma scale, eyes open, never, 24 hours or more after hospital admission
R402120	coma scale, eyes open, to pain, never, unspecified
R402121	coma scale, eyes open, to pain, never, in the field
R402122	coma scale, eyes open, to pain, at arrival to the emergency department
R402123	coma scale, eyes open, to pain, at hospital admission
R402124	coma scale, eyes open, to pain, 24 hours or more after hospital admission
R402210	coma scale, best verbal response, none, unspecified
R402211	coma scale, best verbal response, none, in the field
R402212	coma scale, best verbal response, none, at arrival to the emergency department
R402213	coma scale, best verbal response, none, at hospital admission

R402214	coma scale, best verbal response, none, 24 hours or more after hospital admission
R402220	coma scale, best verbal response, incomprehensible words, unspecified
R402221	coma scale, best verbal response, incomprehensible words, in the field
R402222	coma scale, best verbal response, incomprehensible words, at arrival to the emergency department
R402223	coma scale, best verbal response, incomprehensible words, at hospital admission
R402224	coma scale, best verbal response, incomprehensible words, 24 hours or more after hospital admission
R402310	coma scale, best motor response, none, unspecified
R402311	coma scale, best motor response, none, in the field
R402312	coma scale, best motor response, none, at arrival to the emergency department
R402313	coma scale, best motor response, none, at hospital admission
R402314	coma scale, best motor response, none, 24 hours or more after hospital admission

R402320	coma scale, best motor response, extension, unspecified
R402321	coma scale, best motor response, extension, in the field
R402322	coma scale, best motor response, extension, at arrival to the emergency department
R402323	coma scale, best motor response, extension, at hospital admission
R402324	coma scale, best motor response, extension, 24 hours or more after hospital admission
R402340	coma scale, best motor response, flexion withdrawal, unspecified
R402341	coma scale, best motor response, flexion withdrawal, in the field
R402342	coma scale, best motor response, flexion withdrawal, at arrival to the emergency department
R402343	coma scale, best motor response, flexion withdrawal, at hospital admission
R402344	coma scale, best motor response, flexion withdrawal, 24 hours or more after hospital admission
R402350	coma scale, best motor response, localizes pain, unspecified

R402351	coma scale, best motor response, localizes pain, in the field
R402352	coma scale, best motor response, localizes pain, at arrival to the emergency department
R402353	coma scale, best motor response, localizes pain, at hospital admission
R402354	coma scale, best motor response, localizes pain, 24 hours or more after hospital admission
R402430	Glasgow coma scale score 3-8, unspecified time
R402431	Glasgow coma scale score 3-8, in the field
R402432	Glasgow coma scale score 3-8, at arrival to emergency department
R402433	Glasgow coma scale score 3-8, at hospital admission
R402434	Glasgow coma scale score 3-8, 24 hours or more after hospital admission
R402440	other coma, ,without documented glasgow coma scale score, or with partial score reported, unspecified time
R402441	other coma, ,without documented glasgow coma scale score, or with partial score reported, in the field

R402442	other coma, ,without documented glasgow coma scale score, or with partial score reported, at arrival to emergency department
R402443	other coma, ,without documented glasgow coma scale score, or with partial score reported, at hospital admission
R402444	other coma, ,without documented glasgow coma scale score, or with partial score reported, 24 hours or more after hospital admission
R403	persistent vegetative state
K7041	alcoholic hepatic failure with coma
K7111	toxic liver disease with hepatic necrosis with coma
K72.01	acute and subacute hepatic failure with coma
K72.11	chronic hepatic failure with coma
K72.91	hepatic failure, unspecified, with coma
B150	hepatitis a with hepatic coma
B160	acute hepatitis B with delta agent with hepatic coma

B162	acute hepatitis B without delta agent with hepatic coma
B1711	acute hepatitis C with hepatic coma
B190	unspecified viral hepatitis with hepatic coma
B1911	unspecified viral hepatitis B with hepatic coma
B1921	unspecified viral hepatitis C with hepatic coma
E0801	diametes mellitus due to underlying condition with hyperosmolarity with coma
E0811	Diabetes mellitus due to underlying condition with ketoacidosis with coma
E08641	Diabetes mellitus due to underlying condision with hypoglycemia with coma
E0901	drug or chemical induced diabetes mellitus with hyperosmolarity with coma
E0911	drug or chemical induced diabetes mellitus with ketoacidosis with coma
E09641	drug or chemical induced diabetes mellitus with hypoglycemia with coma

E1011	type 1 diabetes mellitus with ketoacidosis with coma
E10641	type 1 diabetes mellitus with hypoglycemia with coma
E1101	type 2 diabetes mellitus with hyperosmolarity with coma
E11641	type 2 diabetes mellitus with hypoglycemia with coma
E1301	other specified diabetes mellitus with hyperosmolarity with coma
E1311	other specified diabetes mellitus with ketoacidosis with coma
E13641	other specified diabetes mellitus with hypoglycemia with coma
E15	nondiabetic hypoglycemia coma

Alcohol Use ICD10 Codes	
F10	Alcohol Related disorders
F10.1	Alcohol abuse
F10.10	Alcohol abuse uncomplicated
F10.11	Alcohol abuse in remission
F10.12	Alcohol abuse with intoxication
F10.120	Alcohol abuse with intoxication, uncomplicated
F10.121	Alcohol abuse with intoxication, with delirium
F10.129	Alcohol abuse with intoxication, unspecified
F10.13	Alcohol abuse, with withdrawal
F10.130	Alcohol abuse, with withdrawal, uncomplicated
F10.131	Alcohol abuse, with withdrawal delirium
F10.132	Alcohol abuse, with withdrawal with perceptual disturbance
F10.139	Alcohol abuse, with withdrawal, unspecified
F10.14	Alcohol abuse with alcohol induced mood disorder
F10.15	Alcohol abuse with alcohol induced psychotic disorder
F10.150	Alcohol abuse with alcohol induced psychotic disorder, with delusions
F10.151	Alcohol abuse with alcohol induced psychotic disorder, with hallucinations
F10.159	Alcohol abuse with alcohol induced psychotic disorder, unspecied
F10.18	Alcohol abuse with other alcohol induced disorders

F10.180	Alcohol abuse with alcohol induced anxiety disorder
F10.181	Alcohol abuse with alcohol induced sexual dysfunction
F10.182	Alcohol abuse with alcohol induced sleep disorder
F10.188	Alcohol abuse with other alcohol induced disorder
F10.19	Alcohol abuse with unspecified alcohol induced disorder
F10.2	Alcohol dependence
F10.20	Alcohol dependence, uncomplicated
F10.21	Alcohol dependence, in remission
F10.22	Alcohol dependence with intoxication
F10.220	Alcohol dependence with intoxication, uncomplicated
F10.221	Alcohol dependence with intoxication, with delirium
F10.229	Alcohol dependence with intoxication, unspecified
F10.23	Alcohol dependence with withdrawal
F10.230	Alcohol dependence with withdrawal, uncomplicated
F10.231	Alcohol dependence with withdrawal, with delirium
F10.232	Alcohol dependence with withdrawal, with perceptual disturbance
F10.239	Alcohol dependence with withdrawal, unspecified
F10.24	Alcohol dependence with alcohol-induced mood disorder
F10.25	Alcohol dependence with alcohol induced psychotic disorder

F10.250	Alcohol dependence with alcohol induced psychotic disorder with delusions
F10.251	Alcohol dependence with alcohol induced psychotic disorder with hallucinations
F10.259	Alcohol dependence with alcohol induced psychotic disorder unspecified
F10.26	Alcohol dependence with alcohol induced persisting amnestic disorder
F10.27	Alcohol dependence with alcohol induced persisting dementia
F10.28	Alcohol dependence with other alcohol induced disordereds
F10.280	Alcohol dependence with alcohol induced anxiety disorder
F10.281	Alcohol dependence with alcohol induced sexual dysfunction
F10.282	Alcohol dependence with alcohol induced sleep disorder
F10.288	Alcohol dependence with other alcohol induced disorder
F10.29	Alcohol dependence with unspecified alcohol induced disorder
F10.9	alcohol use unspecified
F10.90	Alcohol use , uncomplicated
F10.91	Alcohol use, in remission
F10.92	Alcohol use, unspecified with intoxication
F10.920	Alcohol use, unspecified with intoxication, uncomplicated

F10.921	Alcohol use, unspecified with intoxication, with delirium
F10.929	Alcohol use, unspecified with intoxication , unspecified
F10.93	Alcohol use, unspecified with withdrawal
F10.930	Alcohol use, unspecified with withdrawal, uncomplicated
F10.931	Alcohol use, unspecified with withdrawal, with delirium
F10.932	Alcohol use, unspecified with withdrawal, with perceptual disturbance
F10.939	Alcohol use, unspecified with withdrawal, unspecified
F10.94	Alcohol use, with alcohol induced mood disorder
F10.95	Alcohol use, unspecified with alcohol induced psychotic disorder
F10.950	Alcohol use, unspecified with alcohol induced psychotic disorder with delusions
F10.951	Alcohol use, unspecified with alcohol induced psychotic disorder with hallucinations
F10.959	Alcohol use, unspecified with alcohol induced psychotic disorder, unspecified
F10.96	Alcohol use, unspecified with alcohol induced psychotic disorder with persisteic amnestic disorder

F10.97	Alcohol use, unspecified with alcohol induced psychotic disorder with alcohol induced persisting dementia
F10.98	Alcohol use, unspecified with other alcohol induced disorders
F10.980	Alcohol use, unspecified with alcohol induced anxiety disorder
F10.981	Alcohol use, unspecified with alcohol induced sexual dysfunction
F10.982	Alcohol use, unspecified with alcohol induced sleep disorder
F10.988	Alcohol use, unspecified with other alcohol induced disorder
F10.99	Alcohol use, unspecified with alcohol induced anxiety disorder with unspecified alcohol induced disorder

Opioid Use ICD10 Codes	
F11	Opioid Related disorders
F11.1	Opioid abuse
F11.10	Opioid abuse uncomplicated
F11.11	Opioid abuse in remission
F11.12	Opioid abuse with intoxication
F11.120	Opioid abuse with intoxication, uncomplicated
F11.121	Opioid abuse with intoxication, with delirium
F11.122	Opioid abuse with intoxication, with perceptual disturbance
F11.129	Opioid abuse with intoxication, unspecified
F11.13	Opioid abuse, with withdrawal
F11.14	Opioid abuse with Opioid induced mood disorder
F11.15	Opioid abuse with Opioid induced psychotic disorder
F11.150	Opioid abuse with Opioid induced psychotic disorder, with delusions
F11.151	Opioid abuse with Opioid induced psychotic disorder, with hallucinations
F11.159	Opioid abuse with Opioid induced psychotic disorder, unspecied
F11.18	Opioid abuse with other Opioid induced disorders
F11.181	Opioid abuse with Opioid induced sexual dysfunction

F11.182	Opioid abuse with Opioid induced sleep disorder
F11.188	Opioid abuse with other Opioid induced disorder
F11.19	Opioid abuse with unspecified Opioid induced disorder
F11.2	Opioid dependence
F11.20	Opioid dependence, uncomplicated
F.10.21	Opioid dependence, in remission
F11.22	Opioid dependence with intoxication
F11.220	Opioid dependence with intoxication, uncomplicated
F11.221	Opioid dependence with intoxication, with delirium
F11.222	Opioid Dependence with intoxication, with perceptual disturbance
F11.229	Opioid dependence with intoxication, unspecified
F11.23	Opioid dependence with withdrawal
F11.25	Opioid dependence with opioid induced psychotic disorder
F11.250	Opioid dependence with opioid induced psychotic disorder with delusions
F11.251	Opioid dependence with opioid induced psychotic disorder with hallucinations
F11.259	Opioid dependence with opioid induced psychotic disorder unspecified
F11.28	Opioid dependence with other Opioid induced disorder
F11.281	Opioid dependence with Opioid induced sexual dysfunction

F11.282	Opioid dependence with Opioid induced sleep disorder
F11.288	Opioid dependence with other Opioid induced disorder
F11.29	Opioid dependence with unspecified Opioid induced disorder
F11.9	Opioid use unspecified
F11.90	Opioid use , uncomplicated
F11.91	Opioid use, in remission
F11.92	Opioid use, unspecified with intoxication
F11.920	Opioid use, unspecified with intoxication, uncomplicated
F11.921	Opioid use, unspecified with intoxication, with delirium
F11.922	Opioid use, unspecified with intoxication, with perceptual disturbance
F11.929	Opioid use, unspecified with intoxication , unspecified
F11.93	Opioid use, unspecified with withdrawal
F11.94	Opioid use, with Opioid induced mood disoder
F11.95	Opioid use, unspecified with Opioid induced psychotic disoder
F11.950	Opioid use, unspecified with Opioid induced psychotic disoder with delusions

F11.951	Opioid use, unspecified with Opioid induced psychotic disoder with hallucinations
F11.959	Opioid use, unspecified with Opioid induced psychotic disoder, unspecified
F11.98	Opioid use, unspecified with other Opioid induced disorders
F11.981	Opioid use, unspecified with Opioid induced sexual dysfunction
F11.982	Opioid use, unspecified with Opioid induced sleep disorder
F11.988	Opioid use, unspecified with other Opioid induced disorder
F11.99	Opioid use, unspecified with Opioid induced anxiety disorder with unspecified Opioid induced disorder

Cannabis ICD10	
F12	Cannabis Related disorders
F12.1	Cannabis abuse
F12.10	Cannabis abuse uncomplicated
F12.11	Cannabis abuse in remission
F12.12	Cannabis abuse with intoxication
F12.120	Cannabis abuse with intoxication, uncomplicated
F12.121	Cannabis abuse with intoxication, with delirium
F12.122	Cannabis abuse with intoxication, with perceptual disturbance
F12.129	Cannabis abuse with intoxication, unspecified
F12.13	Cannabis abuse, with withdrawal
F12.15	Cannabis abuse with Cannabis induced psychotic disorder
F12.150	Cannabis abuse with Cannabis induced psychotic disorder, with delusions
F12.151	Cannabis abuse with Cannabis induced psychotic disorder, with hallucinations
F12.159	Cannabis abuse with Cannabis induced psychotic disorder, unspecied
F12.18	Cannabis abuse with other Cannabis induced disorders
F12.180	Cannabis abuse with Cannabis induced anxiety disorder
F12.188	Cannabis abuse with other Cannabis induced disorder
F12.19	Cannabis abuse with unspecified Cannabis induced disorder

F12.2	Cannabis dependence
F12.20	Cannabis dependence, uncomplicated
F.10.21	Cannabis dependence, in remission
F12.22	Cannabis dependence with intoxication
F12.220	Cannabis dependence with intoxication, uncomplicated
F12.221	Cannabis dependence with intoxication, with delirium
F12.229	Cannabis dependence with intoxication, unspecified
F12.23	Cannabis dependence with withdrawal
F12.25	Cannabis dependence with cannabis induced psychotic disorder
F12.250	Cannabis dependence with cannabis induced psychotic disorder with delusions
F12.251	Cannabis dependence with cannabis induced psychotic disorder with hallucinations
F12.259	Cannabis dependence with cannabis induced psychotic disorder unspecified
F12.28	Cannabis dependence with other Cannabis induced disordereds
F12.280	Cannabis dependence with Cannabis induced anxiety disorder

F12.288	Cannabis dependence with other Cannabis induced disorder
F12.29	Cannabis dependence with unspecified Cannabis induced disorder
F12.9	Cannabis use unspecified
F12.90	Cannabis use , uncomplicated
F12.91	Cannabis use, in remission
F12.92	Cannabis use, unspecified with intoxication
F12.920	Cannabis use, unspecified with intoxication, uncomplicated
F12.921	Cannabis use, unspecified with intoxication, with delirium
F12.922	Cannabis use, unspecified with intoxication, with perceptual disturbance
F12.929	Cannabis use, unspecified with intoxication , unspecified
F12.93	Cannabis use, unspecified with withdrawal
F12.95	Cannabis use, unspecified with Cannabis induced psychotic disorder
F12.950	Cannabis use, unspecified with Cannabis induced psychotic disorder with delusions
F12.951	Cannabis use, unspecified with Cannabis induced psychotic disorder with hallucinations

F12.959	Cannabis use, unspecified with Cannabis induced psychotic disorder, unspecified
F12.98	Cannabis use, unspecified with other Cannabis induced disorders
F12.980	Cannabis use, unspecified with Cannabis induced anxiety disorder
F12.988	Cannabis use, unspecified with other Cannabis induced disorder
F12.99	Cannabis use, unspecified with Cannabis induced anxiety disorder with unspecified Cannabis induced disorder

Sedative, Hypnotic, Anxiolytic ICD10 Codes	
F13	sedative, hyponotic, or anxiolytic Related disorders
F13.1	sedative, hyponotic, or anxiolytic abuse
F13.10	sedative, hyponotic, or anxiolytic abuse uncomplicated
F13.11	sedative, hyponotic, or anxiolytic abuse in remission
F13.12	sedative, hyponotic, or anxiolytic abuse with intoxication
F13.120	sedative, hyponotic, or anxiolytic abuse with intoxication, uncomplicated
F13.121	sedative, hyponotic, or anxiolytic abuse with intoxication, with delirium
F13.129	sedative, hyponotic, or anxiolytic abuse with intoxication, unspecified
F13.13	sedative, hyponotic, or anxiolytic abuse, with withdrawal
F13.130	sedative, hyponotic, or anxiolytic abuse, with withdrawal, uncomplicated
F13.131	sedative, hyponotic, or anxiolytic abuse, with withdrawal delirium
F13.132	sedative, hyponotic, or anxiolytic abuse, with withdrawal with perceptual distrubance
F13.139	sedative, hyponotic, or anxiolytic abuse, with withdrawal, unspecified
F13.14	sedative, hyponotic, or anxiolytic abuse with sedative, hyponotic, or anxiolytic induced mood disorder
F13.15	sedative, hyponotic, or anxiolytic abuse with sedative, hyponotic, or anxiolytic induced psychotic disorder

F13.150	sedative, hyponotic, or anxiolytic abuse with sedative, hyponotic, or anxiolytic induced psychotic disorder, with delusions
F13.151	sedative, hyponotic, or anxiolytic abuse with sedative, hyponotic, or anxiolytic induced psychotic disorder, with hallucinations
F13.159	sedative, hyponotic, or anxiolytic abuse with sedative, hyponotic, or anxiolytic induced psychotic disorder, unspecied
F13.18	sedative, hyponotic, or anxiolytic abuse with other sedative, hyponotic, or anxiolytic induced disorders
F13.180	sedative, hyponotic, or anxiolytic abuse with sedative, hyponotic, or anxiolytic induced anxiety disorder
F13.181	sedative, hyponotic, or anxiolytic abuse with sedative, hyponotic, or anxiolytic induced sexual dysfunction
F13.182	sedative, hyponotic, or anxiolytic abuse with sedative, hyponotic, or anxiolytic induced sleep disorder
F13.188	sedative, hyponotic, or anxiolytic abuse with other sedative, hyponotic, or anxiolytic induced disorder
F13.19	sedative, hyponotic, or anxiolytic abuse with unspecified sedative, hyponotic, or anxiolytic induced disorder
F13.2	sedative, hyponotic, or anxiolytic dependence
F13.20	sedative, hyponotic, or anxiolytic dependence, uncomplicated

F.10.21	sedative, hyponotic, or anxiolytic dependence, in remission
F13.22	sedative, hyponotic, or anxiolytic dependence with intoxication
F13.220	sedative, hyponotic, or anxiolytic dependence with intoxication, uncomplicated
F13.221	sedative, hyponotic, or anxiolytic dependence with intoxication, with delirium
F13.229	sedative, hyponotic, or anxiolytic dependence with intoxication, unspecified
F13.23	sedative, hyponotic, or anxiolytic dependence with withdrawal
F13.230	sedative, hyponotic, or anxiolytic dependence with withdrawal, uncomplicated
F13.231	sedative, hyponotic, or anxiolytic dependence with withdrawal, with delirium
F13.232	sedative, hyponotic, or anxiolytic dependence with withdrawal, with perceptual disturbance
F13.239	sedative, hyponotic, or anxiolytic dependence with withdrawal, unspecified
F13.24	sedative, hyponotic, or anxiolytic dependence with sedative, hyponotic, or anxiolytic-induced mood disorder
F13.25	sedative, hyponotic, or anxiolytic dependence with sedative, hyponotic, or anxiolytic induced psychotic disorder
F13.250	sedative, hyponotic, or anxiolytic dependence with sedative, hyponotic, or anxiolytic induced psychotic disorder with delusions

F13.251	sedative, hyponotic, or anxiolytic dependence with sedative, hyponotic, or anxiolytic induced psychotic disorder with hallucinations
F13.259	sedative, hyponotic, or anxiolytic dependence with sedative, hyponotic, or anxiolytic induced psychotic disorder unspecified
F13.26	sedative, hyponotic, or anxiolytic dependence with sedative, hyponotic, or anxiolytic induced persisting amnestic disorder
F13.27	sedative, hyponotic, or anxiolytic dependence with sedative, hyponotic, or anxiolytic induced persisting dementia
F13.28	sedative, hyponotic, or anxiolytic dependence with other sedative, hyponotic, or anxiolytic induced disordereds
F13.280	sedative, hyponotic, or anxiolytic dependence with sedative, hyponotic, or anxiolytic induced anxiety disorder
F13.281	sedative, hyponotic, or anxiolytic dependence with sedative, hyponotic, or anxiolytic induced sexual dysfunction
F13.282	sedative, hyponotic, or anxiolytic dependence with sedative, hyponotic, or anxiolytic induced sleep disorder
F13.288	sedative, hyponotic, or anxiolytic dependence with other sedative, hyponotic, or anxiolytic induced disorder
F13.29	sedative, hyponotic, or anxiolytic dependence with unspecified sedative, hyponotic, or anxiolytic induced disorder

F13.9	sedative, hyponotic, or anxiolytic use unspecified
F13.90	sedative, hyponotic, or anxiolytic use , uncomplicated
F13.91	sedative, hyponotic, or anxiolytic use, in remission
F13.92	sedative, hyponotic, or anxiolytic use, unspecified with intoxication
F13.920	sedative, hyponotic, or anxiolytic use, unspecified with intoxication, uncomplicated
F13.921	sedative, hyponotic, or anxiolytic use, unspecified with intoxication, with delirium
F13.929	sedative, hyponotic, or anxiolytic use, unspecified with intoxication , unspecified
F13.93	sedative, hyponotic, or anxiolytic use, unspecified with withdrawal
F13.930	sedative, hyponotic, or anxiolytic use, unspecified with withdrawal, uncomplicated
F13.931	sedative, hyponotic, or anxiolytic use, unspecified with withdrawal, with delirium
F13.932	sedative, hyponotic, or anxiolytic use, unspecified with withdrawal, with perceptual disturbance
F13.939	sedative, hyponotic, or anxiolytic use, unspecified with withdrawal, unspecified
F13.94	sedative, hyponotic, or anxiolytic use, with sedative, hyponotic, or anxiolytic induced mood disorder
F13.95	sedative, hyponotic, or anxiolytic use, unspecified with sedative, hyponotic, or anxiolytic induced psychotic disorder

F13.950	sedative, hyponotic, or anxiolytic use, unspecified with sedative, hyponotic, or anxiolytic induced psychotic disorder with delusions
F13.951	sedative, hyponotic, or anxiolytic use, unspecified with sedative, hyponotic, or anxiolytic induced psychotic disorder with hallucinations
F13.959	sedative, hyponotic, or anxiolytic use, unspecified with sedative, hyponotic, or anxiolytic induced psychotic disorder, unspecified
F13.96	sedative, hyponotic, or anxiolytic use, unspecified with sedative, hyponotic, or anxiolytic induced psychotic disorder with persisteic amnestic disorder
F13.97	sedative, hyponotic, or anxiolytic use, unspecified with sedative, hyponotic, or anxiolytic induced psychotic disorder with sedative, hyponotic, or anxiolytic induced persisting dementia
F13.98	sedative, hyponotic, or anxiolytic use, unspecified with other sedative, hyponotic, or anxiolytic induced disorders
F13.980	sedative, hyponotic, or anxiolytic use, unspecified with sedative, hyponotic, or anxiolytic induced anxiety disorder
F13.981	sedative, hyponotic, or anxiolytic use, unspecified with sedative, hyponotic, or anxiolytic induced sexual dysfunction
F13.982	sedative, hyponotic, or anxiolytic use, unspecified with sedative, hyponotic, or anxiolytic induced sleep disorder

F13.988	sedative, hyponotic, or anxiolytic use, unspecified with other sedative, hyponotic, or anxiolytic induced disorder
F13.99	sedative, hyponotic, or anxiolytic use, unspecified with sedative, hyponotic, or anxiolytic induced anxiety disorder with unspecified sedative, hyponotic, or anxiolytic induced disorder

Cocaine ICD10 Codes	
F14	Cocaine Related disorders
F14.1	Cocaine abuse
F14.10	Cocaine abuse uncomplicated
F14.11	Cocaine abuse in remission
F14.12	Cocaine abuse with intoxication
F14.120	Cocaine abuse with intoxication, uncomplicated
F14.121	Cocaine abuse with intoxication, with delirium
F14.122	Cocaine abuse with intoxication with perceptual disturbance
F14.129	Cocaine abuse with intoxication, unspecified
F14.13	Cocaine abuse, with withdrawal
F14.14	Cocaine abuse with Cocaine induced mood disorder
F14.15	Cocaine abuse with Cocaine induced psychotic disorder
F14.150	Cocaine abuse with Cocaine induced psychotic disorder, with delusions
F14.151	Cocaine abuse with Cocaine induced psychotic disorder, with hallucinations
F14.159	Cocaine abuse with Cocaine induced psychotic disorder, unspecied
F14.18	Cocaine abuse with other Cocaine induced disorders
F14.180	Cocaine abuse with Cocaine induced anxiety disorder
F14.181	Cocaine abuse with Cocaine induced sexual dysfunction
F14.182	Cocaine abuse with Cocaine induced sleep disorder

F14.188	Cocaine abuse with other Cocaine induced disorder
F14.19	Cocaine abuse with unspecified Cocaine induced disorder
F14.2	Cocaine dependence
F14.20	Cocaine dependence, uncomplicated
F.10.21	Cocaine dependence, in remission
F14.22	Cocaine dependence with intoxication
F14.220	Cocaine dependence with intoxication, uncomplicated
F14.221	Cocaine dependence with intoxication, with delirium
F14.222	Cocaine dependence with intoxication, with perceptual disturbance
F14.229	Cocaine dependence with intoxication, unspecified
F14.23	Cocaine dependence with withdrawal
F14.24	Cocaine dependence with Cocaine-induced mood disorder
F14.25	Cocaine dependence with Cocaine induced psychotic disorder
F14.250	Cocaine dependence with Cocaine induced psychotic disorder with delusions
F14.251	Cocaine dependence with Cocaine induced psychotic disorder with hallucinations
F14.259	Cocaine dependence with Cocaine induced psychotic disorder unspecified
F14.28	Cocaine dependence with other Cocaine induced disordereds
F14.280	Cocaine dependence with Cocaine induced anxiety disorder
F14.281	Cocaine dependence with Cocaine induced sexual dysfunction

F14.282	Cocaine dependence with Cocaine induced sleep disorder
F14.288	Cocaine dependence with other Cocaine induced disorder
F14.29	Cocaine dependence with unspecified Cocaine induced disorder
F14.9	Cocaine use unspecified
F14.90	Cocaine use , uncomplicated
F14.91	Cocaine use, in remission
F14.92	Cocaine use, unspecified with intoxication
F14.920	Cocaine use, unspecified with intoxication, uncomplicated
F14.921	Cocaine use, unspecified with intoxication, with delirium
F14.922	Cocaine use, unspecified with intoxication with perceptual disturbance
F14.929	Cocaine use, unspecified with intoxication , unspecified
F14.93	Cocaine use, unspecified with withdrawal
F14.94	Cocaine use, with Cocaine induced mood disorder
F14.95	Cocaine use, unspecified with Cocaine induced psychotic disorder
F14.950	Cocaine use, unspecified with Cocaine induced psychotic disorder with delusions

F14.951	Cocaine use, unspecified with Cocaine induced psychotic disorder with hallucinations
F14.959	Cocaine use, unspecified with Cocaine induced psychotic disorder, unspecified
F14.98	Cocaine use, unspecified with other Cocaine induced disorders
F14.980	Cocaine use, unspecified with Cocaine induced anxiety disorder
F14.981	Cocaine use, unspecified with Cocaine induced sexual dysfunction
F14.982	Cocaine use, unspecified with Cocaine induced sleep disorder
F14.988	Cocaine use, unspecified with other Cocaine induced disorder
F14.99	Cocaine use, unspecified with Cocaine induced anxiety disorder with unspecified Cocaine induced disorder

Stimulant ICD10 Codes	
F15	Other stimulant Related disorders
F15.1	Other stimulant abuse
F15.10	Other stimulant abuse uncomplicated
F15.11	Other stimulant abuse in remission
F15.12	Other stimulant abuse with intoxication
F15.120	Other stimulant abuse with intoxication, uncomplicated
F15.121	Other stimulant abuse with intoxication, with delirium
F15.122	Other stimulant abuse with intoxication with perceptual disturbance
F15.129	Other stimulant abuse with intoxication, unspecified
F15.13	Other stimulant abuse, with withdrawal
F15.14	Other stimulant abuse with Other stimulant induced mood disorder
F15.15	Other stimulant abuse with Other stimulant induced psychotic disorder
F15.150	Other stimulant abuse with Other stimulant induced psychotic disorder, with delusions
F15.151	Other stimulant abuse with Other stimulant induced psychotic disorder, with hallucinations
F15.159	Other stimulant abuse with Other stimulant induced psychotic disorder, unspecied
F15.18	Other stimulant abuse with other Other stimulant induced disorders

F15.180	Other stimulant abuse with Other stimulant induced anxiety disorder
F15.181	Other stimulant abuse with Other stimulant induced sexual dysfunction
F15.182	Other stimulant abuse with Other stimulant induced sleep disorder
F15.188	Other stimulant abuse with other Other stimulant induced disorder
F15.19	Other stimulant abuse with unspecified Other stimulant induced disorder
F15.2	Other stimulant dependence
F15.20	Other stimulant dependence, uncomplicated
F.10.21	Other stimulant dependence, in remission
F15.22	Other stimulant dependence with intoxication
F15.220	Other stimulant dependence with intoxication, uncomplicated
F15.221	Other stimulant dependence with intoxication, with delirium
F15.222	Other stimulant dependence with intoxication, with perceptual disturbance
F15.229	Other stimulant dependence with intoxication, unspecified
F15.23	Other stimulant dependence with withdrawal
F15.24	Other stimulant dependence with Other stimulant-induced mood disorder

F15.25	Other stimulant dependence with Other stimulant induced psychotic disorder
F15.250	Other stimulant dependence with Other stimulant induced psychotic disorder with delusions
F15.251	Other stimulant dependence with Other stimulant induced psychotic disorder with hallucinations
F15.259	Other stimulant dependence with Other stimulant induced psychotic disorder unspecified
F15.28	Other stimulant dependence with other Other stimulant induced disordereds
F15.280	Other stimulant dependence with Other stimulant induced anxiety disorder
F15.281	Other stimulant dependence with Other stimulant induced sexual dysfunction
F15.282	Other stimulant dependence with Other stimulant induced sleep disorder
F15.288	Other stimulant dependence with other Other stimulant induced disorder
F15.29	Other stimulant dependence with unspecified Other stimulant induced disorder
F15.9	Other stimulant use unspecified
F15.90	Other stimulant use , uncomplicated
F15.91	Other stimulant use, in remission
F15.92	Other stimulant use, unspecified with intoxication

F15.920	Other stimulant use, unspecified with intoxication, uncomplicated
F15.921	Other stimulant use, unspecified with intoxication, with delirium
F15.922	Other stimulant use, unspecified with intoxication with perceptual disturbance
F15.929	Other stimulant use, unspecified with intoxication , unspecified
F15.93	Other stimulant use, unspecified with withdrawal
F15.94	Other stimulant use, with Other stimulant induced mood disorder
F15.95	Other stimulant use, unspecified with Other stimulant induced psychotic disorder
F15.950	Other stimulant use, unspecified with Other stimulant induced psychotic disorder with delusions
F15.951	Other stimulant use, unspecified with Other stimulant induced psychotic disorder with hallucinations
F15.959	Other stimulant use, unspecified with Other stimulant induced psychotic disorder, unspecified
F15.98	Other stimulant use, unspecified with other Other stimulant induced disorders
F15.980	Other stimulant use, unspecified with Other stimulant induced anxiety disorder
F15.981	Other stimulant use, unspecified with Other stimulant induced sexual dysfunction

F15.982	Other stimulant use, unspecified with Other stimulant induced sleep disorder
F15.988	Other stimulant use, unspecified with other Other stimulant induced disorder
F15.99	Other stimulant use, unspecified with Other stimulant induced anxiety disorder with unspecified Other stimulant induced disorder

Hallucinogen Use ICD10 Codes	
F16	Hallucinogen Related disorders
F16.1	Hallucinogen abuse
F16.10	Hallucinogen abuse uncomplicated
F16.11	Hallucinogen abuse in remission
F16.12	Hallucinogen abuse with intoxication
F16.120	Hallucinogen abuse with intoxication, uncomplicated
F16.121	Hallucinogen abuse with intoxication, with delirium
F16.122	Hallucinogen abuse with intoxication with perceptual disturbance
F16.129	Hallucinogen abuse with intoxication, unspecified
F16.14	Hallucinogen abuse with Hallucinogen induced mood disorder
F16.15	Hallucinogen abuse with Hallucinogen induced psychotic disorder
F16.150	Hallucinogen abuse with Hallucinogen induced psychotic disorder, with delusions
F16.151	Hallucinogen abuse with Hallucinogen induced psychotic disorder, with hallucinations
F16.159	Hallucinogen abuse with Hallucinogen induced psychotic disorder, unspecied
F16.18	Hallucinogen abuse with other Hallucinogen induced disorders
F16.180	Hallucinogen abuse with Hallucinogen induced anxiety disorder

F16.183	Hallucinogen abuse with Hallucinogen persisting perception disorder (flashbacks)
F16.188	Hallucinogen abuse with other Hallucinogen induced disorder
F16.19	Hallucinogen abuse with unspecified Hallucinogen induced disorder
F16.2	Hallucinogen dependence
F16.20	Hallucinogen dependence, uncomplicated
F.10.21	Hallucinogen dependence, in remission
F16.22	Hallucinogen dependence with intoxication
F16.220	Hallucinogen dependence with intoxication, uncomplicated
F16.221	Hallucinogen dependence with intoxication, with delirium
F16.229	Hallucinogen dependence with intoxication, unspecified
F16.24	Hallucinogen dependence with Hallucinogen-induced mood disorder
F16.25	Hallucinogen dependence with Hallucinogen induced psychotic disorder
F16.250	Hallucinogen dependence with Hallucinogen induced psychotic disorder with delusions
F16.251	Hallucinogen dependence with Hallucinogen induced psychotic disorder with hallucinations

F16.259	Hallucinogen dependence with Hallucinogen induced psychotic disorder unspecified
F16.28	Hallucinogen dependence with other Hallucinogen induced disordereds
F16.280	Hallucinogen dependence with Hallucinogen induced anxiety disorder
F16.283	Hallucinogen dependence with Hallucinogen persisting perception disorder (flashbacks)
F16.288	Hallucinogen dependence with other Hallucinogen induced disorder
F16.29	Hallucinogen dependence with unspecified Hallucinogen induced disorder
F16.9	Hallucinogen use unspecified
F16.90	Hallucinogen use , uncomplicated
F16.91	Hallucinogen use, in remission
F16.92	Hallucinogen use, unspecified with intoxication
F16.920	Hallucinogen use, unspecified with intoxication, uncomplicated
F16.921	Hallucinogen use, unspecified with intoxication, with delirium
F16.929	Hallucinogen use, unspecified with intoxication , unspecified
F16.94	Hallucinogen use, with Hallucinogen induced mood disorder

F16.95	Hallucinogen use, unspecified with Hallucinogen induced psychotic disorder
F16.950	Hallucinogen use, unspecified with Hallucinogen induced psychotic disorder with delusions
F16.951	Hallucinogen use, unspecified with Hallucinogen induced psychotic disorder with hallucinations
F16.959	Hallucinogen use, unspecified with Hallucinogen induced psychotic disorder, unspecified
F16.98	Hallucinogen use, unspecified with other Hallucinogen induced disorders
F16.980	Hallucinogen use, unspecified with Hallucinogen induced anxiety disorder
F16.983	Hallucinogen use, unspecified with Hallucinogen persisting perception disorder (flashbacks)
F16.988	Hallucinogen use, unspecified with other Hallucinogen induced disorder
F16.99	Hallucinogen use, unspecified with Hallucinogen induced anxiety disorder with unspecified Hallucinogen induced disorder

Nicotine Use ICD10 Codes	
F17	Nicotine Dependence
F17.2	Nicotine Dependence
F17.20	Nicotine Dependence, unspecified
F17.200	Nicotine Dependence, unspecified uncomplicated
F17.201	Nicotine Dependence, unspecified in remission
F17.203	Nicotine Dependence, unspecified, with withdrawal
F17.208	Nicotine Dependence, unspecified, with withrdrawal with other nicotine induced disorders
F17.209	Nicotine Dependence, unspecified, with
F17.21	Nicotine Dependence, cigarettes
F17.210	Nicotine Dependence, cigarettes, uncomplicated
F17.211	Nicotine Dependence, cigarettes, in remission
F17.213	Nicotine Dependence, cigarettes, with withdrawal
F17.218	Nicotine Dependence, cigarettes, with other nicotine-induced disorders
F17.219	Nicotine Dependence, cigarettes, with unspecified nicotine induced disorders
F17.22	Nicotine Dependence, chewing tobacco
F17.220	Nicotine Dependence, chewing tobacco, uncomplicated
F17.221	Nicotine Dependence, chewing tobacco, in remission
F17.223	Nicotine Dependence, chewing tobacco, with withdrawal

F17.228	Nicotine Dependence, chewing tobacco, with other nicotine-induced disorders
F17.229	Nicotine Dependence, chewing tobacco, with unspecified nicotine induced disorders
F17.29	Nicotine Dependence, other tobacco product
F17.290	Nicotine Dependence, other tobacco product, uncomplicated
F17.291	Nicotine Dependence, other tobacco product, in remission
F17.293	Nicotine Dependence, other tobacco product, with withdrawal
F17.298	Nicotine Dependence, other tobacco product, with other nicotine-induced disorders
F17.299	Nicotine Dependence, other tobacco product, with unspecified nicotine induced disorders

Inhalant Use ICD10 Codes	
F18	Inhalant Related disorders
F18.1	Inhalant abuse
F18.10	Inhalant abuse uncomplicated
F18.11	Inhalant abuse in remission
F18.12	Inhalant abuse with intoxication
F18.120	Inhalant abuse with intoxication, uncomplicated
F18.121	Inhalant abuse with intoxication, with delirium
F18.129	Inhalant abuse with intoxication, unspecified
F18.14	Inhalant abuse with Inhalant induced mood disorder
F18.15	Inhalant abuse with Inhalant induced psychotic disorder
F18.150	Inhalant abuse with Inhalant induced psychotic disorder, with delusions
F18.151	Inhalant abuse with Inhalant induced psychotic disorder, with hallucinations
F18.159	Inhalant abuse with Inhalant induced psychotic disorder, unspecied
F18.17	Inhalant abuse with inhalant induced dementia
F18.18	Inhalant abuse with other Inhalant induced disorders
F18.180	Inhalant abuse with Inhalant induced anxiety disorder
F18.188	Inhalant abuse with other Inhalant induced disorder

F18.19	Inhalant abuse with unspecified Inhalant induced disorder
F18.2	Inhalant dependence
F18.20	Inhalant dependence, uncomplicated
F.10.21	Inhalant dependence, in remission
F18.22	Inhalant dependence with intoxication
F18.220	Inhalant dependence with intoxication, uncomplicated
F18.221	Inhalant dependence with intoxication, with delirium
F18.229	Inhalant dependence with intoxication, unspecified
F18.24	Inhalant dependence with Inhalant-induced mood disorder
F18.25	Inhalant dependence with Inhalant induced psychotic disorder
F18.250	Inhalant dependence with Inhalant induced psychotic disorder with delusions
F18.251	Inhalant dependence with Inhalant induced psychotic disorder with hallucinations
F18.259	Inhalant dependence with Inhalant induced psychotic disorder unspecified
F18.27	Inhalant dependence with inhalant induced dementia
F18.28	Inhalant dependence with other Inhalant induced disordereds

F18.280	Inhalant dependence with Inhalant induced anxiety disorder
F18.288	Inhalant dependence with other Inhalant induced disorder
F18.29	Inhalant dependence with unspecified Inhalant induced disorder
F18.9	Inhalant use unspecified
F18.90	Inhalant use , uncomplicated
F18.91	Inhalant use, in remission
F18.92	Inhalant use, unspecified with intoxication
F18.920	Inhalant use, unspecified with intoxication, uncomplicated
F18.921	Inhalant use, unspecified with intoxication, with delirium
F18.929	Inhalant use, unspecified with intoxication , unspecified
F18.94	Inhalant use, with Inhalant induced mood disorder
F18.95	Inhalant use, unspecified with Inhalant induced psychotic disorder
F18.950	Inhalant use, unspecified with Inhalant induced psychotic disorder with delusions
F18.951	Inhalant use, unspecified with Inhalant induced psychotic disorder with hallucinations

F18.959	Inhalant use, unspecified with Inhalant induced psychotic disorder, unspecified
F18.97	Inhalant Use, unspecified with inhalant induced persisting dementia
F18.98	Inhalant use, unspecified with other Inhalant induced disorders
F18.980	Inhalant use, unspecified with Inhalant induced anxiety disorder
F18.988	Inhalant use, unspecified with other Inhalant induced disorder
F18.99	Inhalant use, unspecified with Inhalant induced anxiety disorder with unspecified Inhalant induced disorder

Other Psychoactive Substance Use ICD10 Codes	
F19	Other psychoactive substance Related disorders
F19.1	Other psychoactive substance abuse
F19.10	Other psychoactive substance abuse uncomplicated
F19.11	Other psychoactive substance abuse in remission
F19.12	Other psychoactive substance abuse with intoxication
F19.120	Other psychoactive substance abuse with intoxication, uncomplicated
F19.121	Other psychoactive substance abuse with intoxication, with delirium
F19.122	Other psychoactive substance abuse with intoxication, with perceptual disturbances
F19.129	Other psychoactive substance abuse with intoxication, unspecified
F19.13	Other psychoactive substance abuse, with withdrawal
F19.130	Other psychoactive substance abuse, with withdrawal, uncomplicated
F19.131	Other psychoactive substance abuse, with withdrawal delirium
F19.132	Other psychoactive substance abuse, with withdrawal with perceptual distrubance
F19.139	Other psychoactive substance abuse, with withdrawal, unspecified
F19.14	Other psychoactive substance abuse with Other psychoactive substance induced mood disorder
F19.15	Other psychoactive substance abuse with Other psychoactive substance induced psychotic disorder
F19.150	Other psychoactive substance abuse with Other psychoactive substance induced psychotic disorder, with delusions
F19.151	Other psychoactive substance abuse with Other psychoactive substance induced psychotic disorder, with hallucinations

F19.159	Other psychoactive substance abuse with Other psychoactive substance induced psychotic disorder, unspecied
F19.16	Other psychoactive substance abuse with psychoactive substance-induced persisting amnestic disorder
F19.17	Other psychoactive substance abuse with psychoactive substance induced persisting dementia
F19.18	Other psychoactive substance abuse with other Other psychoactive substance induced disorders
F19.180	Other psychoactive substance abuse with Other psychoactive substance induced anxiety disorder
F19.181	Other psychoactive substance abuse with Other psychoactive substance induced sexual dysfunction
F19.182	Other psychoactive substance abuse with Other psychoactive substance induced sleep disorder
F19.188	Other psychoactive substance abuse with other Other psychoactive substance induced disorder
F19.19	Other psychoactive substance abuse with unspecified Other psychoactive substance induced disorder
F19.2	Other psychoactive substance dependence
F19.20	Other psychoactive substance dependence, uncomplicated
F.10.21	Other psychoactive substance dependence, in remission
F19.22	Other psychoactive substance dependence with intoxication
F19.220	Other psychoactive substance dependence with intoxication, uncomplicated
F19.221	Other psychoactive substance dependence with intoxication, with delirium
F19.2222	Other psychoactive substance dependence with intoxication with perceptual disturbance

F19.229	Other psychoactive substance dependence with intoxication, unspecified
F19.23	Other psychoactive substance dependence with withdrawal
F19.230	Other psychoactive substance dependence with withdrawal, uncomplicated
F19.231	Other psychoactive substance dependence with withdrawal, with delirium
F19.232	Other psychoactive substance dependence with withdrawal, with perceptual disturbance
F19.239	Other psychoactive substance dependence with withdrawal, unspecified
F19.24	Other psychoactive substance dependence with Other psychoactive substance-induced mood disorder
F19.25	Other psychoactive substance dependence with Other psychoactive substance induced psychotic disorder
F19.250	Other psychoactive substance dependence with Other psychoactive substance induced psychotic disorder with delusions
F19.251	Other psychoactive substance dependence with Other psychoactive substance induced psychotic disorder with hallucinations
F19.259	Other psychoactive substance dependence with Other psychoactive substance induced psychotic disorder unspecified
F19.26	Other psychoactive substance dependence with Other psychoactive substance induced persisting amnestic disorder
F19.27	Other psychoactive substance dependence with Other psychoactive substance induced persisting dementia
F19.28	Other psychoactive substance dependence with other Other psychoactive substance induced disordereds

F19.280	Other psychoactive substance dependence with Other psychoactive substance induced anxiety disorder
F19.281	Other psychoactive substance dependence with Other psychoactive substance induced sexual dysfunction
F19.282	Other psychoactive substance dependence with Other psychoactive substance induced sleep disorder
F19.288	Other psychoactive substance dependence with other Other psychoactive substance induced disorder
F19.29	Other psychoactive substance dependence with unspecified Other psychoactive substance induced disorder
F19.9	Other psychoactive substance use unspecified
F19.90	Other psychoactive substance use , uncomplicated
F19.91	Other psychoactive substance use, in remission
F19.92	Other psychoactive substance use, unspecified with intoxication
F19.920	Other psychoactive substance use, unspecified with intoxication, uncomplicated
F19.921	Other psychoactive substance use, unspecified with intoxication, with delirium
F19.922	Other psychoactive substance use, unspecified with intoxication, with perceptual disturbance
F19.929	Other psychoactive substance use, unspecified with intoxication , unspecified
F19.93	Other psychoactive substance use, unspecified with withdrawal
F19.930	Other psychoactive substance use, unspecified with withdrawal, uncomplicated
F19.931	Other psychoactive substance use, unspecified with withdrawal, with delirium

F19.932	Other psychoactive substance use, unspecified with withdrawal, with perceptual disturbance
F19.939	Other psychoactive substance use, unspecified with withdrawal, unspecified
F19.94	Other psychoactive substance use, with Other psychoactive substance induced mood disorder
F19.95	Other psychoactive substance use, unspecified with Other psychoactive substance induced psychotic disorder
F19.950	Other psychoactive substance use, unspecified with Other psychoactive substance induced psychotic disorder with delusions
F19.951	Other psychoactive substance use, unspecified with Other psychoactive substance induced psychotic disorder with hallucinations
F19.959	Other psychoactive substance use, unspecified with Other psychoactive substance induced psychotic disorder, unspecified
F19.96	Other psychoactive substance use, unspecified with Other psychoactive substance induced psychotic disorder with persisteic amnestic disorder
F19.97	Other psychoactive substance use, unspecified with Other psychoactive substance induced psychotic disorder with Other psychoactive substance induced persisting dementia
F19.98	Other psychoactive substance use, unspecified with other Other psychoactive substance induced disorders
F19.980	Other psychoactive substance use, unspecified with Other psychoactive substance induced anxiety disorder
F19.981	Other psychoactive substance use, unspecified with Other psychoactive substance induced sexual dysfunction

F19.982	Other psychoactive substance use, unspecified with Other psychoactive substance induced sleep disorder
F19.988	Other psychoactive substance use, unspecified with other Other psychoactive substance induced disorder
F19.99	Other psychoactive substance use, unspecified with Other psychoactive substance induced anxiety disorder with unspecified Other psychoactive substance induced disorder

Table 1: Substance use and delirium/coma in the Medical Intensive Care Unit from 2018-2022

	total group	substance use	delirium or coma	substance use with delirium or coma	substance use without delirium or coma	no substance use with delirium or coma	no substance use without delirium or coma
2018	1710	564 (33.3%)	858 (50.2%)	320 (18.7%)	255 (14.9%)	545 (31.9%)	663 (38.8%)
2019	1614	526 (32.6%)	832 (51.5%)	327 (20.3%)	216 (13.4%)	507 (38.6%)	623 (38.6%)
2020	1642	458 (27.9%)	883 (53.8%)	282 (17.2%)	181 (11.0%)	609 (37.8%)	621 (37.8%)
2021	1594	423 (26.5%)	800 (50.2%)	247 (15.5%)	180 (11.3%)	557 (39.9%)	636 (39.9%)
2022	1410	447 (31.7%)	658 (46.7%)	231 (16.4%)	224 (15.9%)	432 (39.6%)	559 (39.6%)

Table 2: Substance use and delirium/coma in the Medical Intensive Care Unit from 2018-2022--Males

	total group	substance use	delirium or coma	substance use with delirium or coma	substance use without delirium or coma	no substance use with delirium or coma	no substance use without delirium or coma
2018	870	326 (37.5%)	455 (52.3%)	194 (22.3%)	137 (15.7%)	263 (30.2%)	354 (40.7%)
2019	801	302 (37.7%)	400 (49.9%)	195 (24.3%)	116 (14.5%)	206 (25.7%)	311 (38.8%)
2020	900	289 (32.1%)	485 (53.9%)	180 (20.0%)	110 (12.2%)	309 (34.3%)	321 (35.7%)
2021	833	248 (29.8%)	409 (49.1%)	140 (16.8%)	110 (13.2%)	272 (32.7%)	321 (38.5%)
2022	710	261 (36.8%)	334 (47.0%)	139 (19.6%)	126 (17.7%)	198 (27.9%)	263 (37.0%)

Table 3: Substance use and delirium/coma in the Medical Intensive Care Unit from 2018-2022--Females

	total group	substance use	delirium or coma	substance use with delirium or coma	substance use without delirium or coma	no substance use with delirium or coma	no substance use without delirium or coma
2018	840	238 (28.3%)	403 (48.0%)	126 (15.0%)	118 (14.0%)	282 (33.6%)	309 (36.8%)
2019	813	224 (27.6%)	432 (53.1%)	132 (16.2%)	100 (12.3%)	301 (37.0%)	312 (38.4%)
2020	742	169 (18.8%)	398 (53.6%)	102 (11.3%)	71 (7.89%)	300 (33.3%)	300 (33.3%)
2021	761	175 (23.0%)	391 (51.4%)	107 (14.1%)	70 (9.20%)	285 (37.5%)	315 (41.4%)
2022	700	186 (26.6%)	324 (46.3%)	92 (13.1%)	98 (14.0%)	234 (33.4%)	296 (42.3%)

Table 4: Substance use and delirium/coma on general medicine floors from 2018-2022

	total group	substance use	delirium or coma	substance use with delirium or coma	substance use without delirium or coma	no substance use with delirium or coma	no substance use without delirium or coma
2018	57546	15054 (26.2%)	7701 (13.4%)	2418 (4.20%)	13225 (23.0%)	5393 (9.37%)	40921 (71.1%)
2019	60282	15715 (26.1%)	8423 (14.0%)	2531 (4.20%)	13952 (23.1%)	6021 (10.0%)	43064 (71.4%)
2020	54675	13327 (24.4%)	8317 (15.2%)	2499 (4.57%)	11560 (21.1%)	5921 (10.8%)	39182 (71.7%)
2021	63472	15072 (23.7%)	9023 (14.2%)	2682 (4.23%)	13121 (20.7%)	6470 (10.2%)	46086 (72.6%)
2022	73233	18665 (25.5%)	10263 (14.0%)	3063 (4.18%)	16436 (22.4%)	7362 (10.1%)	52479 (71.7%)

Table 5: Substance use and delirium/coma on general medicine floors from 2018-2022 – Males

	total group	substance use	delirium or coma	substance use with delirium or coma	substance use without delirium or coma	no substance use with delirium or coma	no substance use without delirium or coma
2018	28087	8687 (30.9%	3685 (13.1%)	1393 (4.96%)	7628 (27.2%	2349 (8.36%)	18958 (67.5%)
2019	29129	9042 (31.0%)	3912 (13.4%)	1457 (5.00%)	8012 (27.5%)	2521 (8.65%)	19736 (67.8%)
2020	27312	7953 (29.1%)	4139 (15.2%)	1512 (5.54%)	6854 (25.1%)	2681 (9.82%)	18589 (68.1%)
2021	31399	8877 (28.3%)	4385 (14.0%)	1585 (5.05%)	7715 (24.6%)	2868 (9.13%)	21744 (69.3%)
2022	35877	10802 (30.1%)	4963 (13.8%)	1769 (4.93%)	9519 (26.5%)	3276 (9.13%)	24435 (68.1%)

Table 6: Substance use and delirium/coma on general medicine floors from 2018-2022 – Females

	total group	substance use	delirium or coma	substance use with delirium or coma	substance use without delirium or coma	no substance use with delirium or coma	no substance use without delirium or coma
2018	29459	6367 (21.6%)	4016 (13.6%)	1025 (3.48%)	5597 (19.0%)	3044 (10.3%)	21963 (74.6%)
2019	31153	6673 (21.4%)	4511 (14.5%)	1074 (3.45%)	5940 (19.1%)	3500 (11.2%)	23328 (74.9%)
2020	27363	5374 (19.7%)	4178 (15.3%)	987 (3.61%)	4706 (17.2%)	3240 (11.9%)	20593 (75.4%)
2021	32073	6195 (19.3%)	4638 (14.5%)	1097 (3.42%)	5406 (16.9%)	3602 (11.2%)	24342 (75.9%)
2022	37356	7863 (21.0%)	5300 (14.2%)	1294 (3.46%)	6917 (18.5%)	4086 (10.9%)	28044 (75.1%)
